# Transported Entropy of Ions and Peltier Coefficients in 8YSZ and 10Sc1CeSZ Electrolytes for Solid Oxide Cells

**DOI:** 10.3390/e26100872

**Published:** 2024-10-17

**Authors:** Aydan Gedik, Stephan Kabelac

**Affiliations:** Institute of Thermodynamics, Leibniz University Hannover, Welfengarten 1, D-30167 Hannover, Germany; kabelac@ift.uni-hannover.de

**Keywords:** solid oxide cell (SOC), non-equilibrium thermodynamics (NET), Seebeck coefficient, Peltier coefficient, transported entropy of ions, electrodes heat generation, reversible heat effects

## Abstract

In this study, the transported entropy of ions for 8YSZ and 10Sc1CeSZ electrolytes was experimentally determined to enable precise modeling of heat transport in solid oxide cells (SOCs). The Peltier coefficient, crucial for thermal management, was directly calculated, highlighting reversible heat transport effects in the cell. While data for 8YSZ are available in the literature, providing a basis for comparison, the results for 10Sc1CeSZ show slightly smaller Seebeck coefficients but higher transported ion entropies. Specifically, at 700°C and an oxygen partial pressure of $p_{O_{2}}=0.21$ bar, values of $S_{O^{2-}}^{*}=52\pm 10$ J/K·F for 10Sc1CeSZ and $S_{O^{2-}}^{*}=48\pm 9$ J/K·F for 8YSZ were obtained. The transported entropy was also validated through theoretical calculations and showed minimal deviations when comparing different cell operation modes (O_2_||O^2−^||O_2_ and H_2_, H_2_O||O^2−^||O_2_). The influence of the transported entropy of the ions on the total heat generation and the partial heat generation at the electrodes is shown. The temperature has the greatest influence on heat generation, whereby the ion entropy also plays a role. Finally, the Peltier coefficients of 8YSZ for all homogeneous phases agree with the literature values.

## 1. Introduction

For cell development and for the optimisation of operating strategies in solid oxide cells (SOCs), a reliable understanding of the heat, mass and charge transport mechanisms is of great importance. In particular, the performance and lifetime of SOCs are influenced by the temperature distribution. Uncontrolled temperature changes can lead to local overheating or undercooling and thus to high-temperature gradients within a cell. A comprehensive literature review on thermal management in SOFC stacks shows that excessive temperature gradients can lead to delamination and cracks in the electrolyte and electrodes [1]. For this reason, it is necessary to recognise not only the amount of total heat in the cell but also the local generation and/or local absorption of heat in all parts of the cell. A systematic establishment of heat balance equations and assessment of transport mechanisms can provide information.

The heat is divided into two types. On the one hand, *irreversible* heat is caused by dissipated energy (ohmic resistances and overvoltages at the electrodes among other sources). On the other hand, *reversible* heat is due to the reaction entropies at the electrodes. The electrochemical reaction at the three-phase interface leads to a Peltier effect, in which Peltier heat is reversibly released or absorbed at each electrode. Using the non-equilibrium thermodynamics (NET) approach, the influence of the Peltier heat can be clearly determined. According to NET, the following equation applies to the heat transport of a homogeneous phase *i* [2]:

(1) $$J_{q}^{i}=-\lambda^{i}\cdot \frac{dT}{dy}+\frac{\pi^{i}}{F}\cdot j,$$

where $\lambda^{i}$ is the thermal conductivity, $\pi^{i}$ is the Peltier coefficient of the homogen phase *i* and *j* is the current density. According to the monocausal approach, the first part of the right-hand side of the equation describes the heat transport induced by a temperature gradient (Fourier heat). The second part of the right-hand side of the equation describes the Peltier heat induced by a charge transport. The importance of the Peltier effect is emphasised by means of a 1D SOFC model with an 8YSZ electrolyte that a heat flux flows in the direction of the temperature maximum near the cathode reaction layer [3]. In comparison, another investigation using a 2D SOEC model with an 8YSZ electrolyte shows that the Peltier effect is more extensive and more pronounced in SOEC operation than in SOFC operation [4]. In both studies, the data for the Peltier coefficients for the homogeneous phases are taken from the literature. Precise knowledge of the Peltier coefficients is essential for a realistic representation of heat transport and thus successful heat management. Compared to the homogeneous phase of the electrolyte, however, the Peltier coefficients for the homogeneous phases of the anode and cathode can be calculated simply.

Peltier coefficients are not directly measurable variables. However, they can be determined via the direct measurement of Seebeck coefficients. If an electrical charge transport occurs due to an imposed temperature gradient, this effect is referred to as the thermoelectric effect or the Seebeck effect. The type of non-isothermal electrochemical cells are referred to as thermocells. A direct relationship between the Peltier coefficient π and the Seebeck coefficient $\alpha_{S}$ can be established via Onsager’s reciprocity relationship [2]:

(2) $$\alpha_{S}={\left(\frac{d\phi }{dT}\right)}_{j\to 0,\;dT\to 0}=-\frac{\pi }{F\cdot T}.$$

The measuring method is based on recording the change in cell potential in an open circuit when a temperature gradient is applied. An electrochemical cell with identical electrodes is considered, in each of which the back-and-forth reaction of an electrochemical reaction takes place.

The determination of Seebeck coefficients of electrochemical converters is common in the literature. Investigations into the Seebeck coefficient are carried out primarily in the field of thermoelectric power generators. Thermogalvanic cells can be used to generate electricity directly from waste heat. In the works [5,6,7,8,9], examples of possible electrochemical converters that can be used for such an objective are considered. Also, with the aim of achieving optimum thermal management, reversible thermal effects for relevant electrodes of different compositions and lithiation levels with different electrolytes are being investigated in the field of lithium-ion batteries. A comprehensive overview can be found in the publication by Gunnarshaug et al. [10]. Supported by previous investigations, the significance of the Peltier heat of a single electrode with regard to the entire cell is also clarified there. With the same aim, the works [11,12,13,14,15,16,17] investigate the reversible heat effects of other electrochemical half-cell reactions that are used in fuel cell and electrolysis technology and classified in the literature.

There are several studies in the literature on experimental work on oxygen-concentration cells with solid oxide electrolytes in the high-temperature range. Kiukkola and Wagner [18] showed by electromotive force (EMF) measurements that ZrO_2_-CaO electrolytes exhibit stable ionic conduction at a temperature of 870 °C over a wide range of atmospheric oxygen partial pressures ($10^{-5}$ to 1 atm), with electronic conduction contributions being minimal. Further studies were then carried out on the ionic and electronic conductivity of solid oxide electrolytes in other oxygen partial pressure and temperature ranges [19,20,21,22,23,24]. By adjusting the H_2_/H_2_O and CO/CO_2_ mixtures, the oxygen activity could be precisely regulated, thus ensuring a reducing atmosphere. Park et al. [25] established empirical correlations for the conductivity of ions, electrons and holes for 8YSZ as a function of temperature and oxygen partial pressure. The ionic conductivity of oxide ceramics is strongly dependent on the temperature, the doping and the dopant. For example, Kosacki et al. [26] show that ionic conduction for ScSZ predominates more in other oxygen partial pressure ranges than it does for 8YSZ.

Fundamental theoretical insights into the behavior of the thermocells and important phenomenological equations for describing their properties are provided by the work of Holtan et al. [27]. With the knowledge gained from the studies on the stability of oxide ceramics over wide temperature and oxygen partial pressure ranges, numerous investigations were undertaken with regard to the thermopower of oxide ceramics with the aim of obtaining more precise information on the mobility of the oxygen ion and its dependencies. Table 1 summarises some values for the Seebeck coefficients of YSZ and ScSZ [28]. Further studies with CSZ can be found, for example, in [24,29,30,31]. The results show that the Seebeck coefficient is clearly dependent on the oxygen partial pressure of the surrounding atmosphere and the concentration of oxygen vacancies in the mixed oxide. Furthermore, there is no clear dependence on temperature [32]. The NET approach makes it possible to calculate the transported ion entropy and the heat of transfer coupled to it; so, it can be concluded from this information that the transport of the ions does not take place via interstitial sites but via vacancies. From this, it was possible to show that the transported ion entropy is independent of the oxygen partial pressure. Ratkje et al. describe the temperature dependence via the Thomson coefficient of the ion [33].

In the work of Takekara et al. [34] and Kanamura et al. [36], the measurement of the Seebeck coefficient of 8YSZ is used for the entropy changes and the associated heat generation of the individual electrodes. Using measurements and empirical correlations, they show how the entropy change in the half-cell reactions behaves as a function of the partial pressure of the hydrogen and oxygen in order to ensure optimisation of the energy efficiency in solid oxide fuel cells through targeted control of this entropy change. The work of Ratkje et al. [33] shows slightly different results, which determines a greater asymmetry in the heat production of the half-cell reactions. The discrepancy is explained by the value of the transported ion entropy, which can have a major influence on the amount of heat produced by the individual electrodes and can even determine whether the anode-side half-cell reaction is exothermic or endothermic. Based on these results, Fischer et al. [37] use a spatially discretised stationary model of a tubular SOFC to show how relevant the influence of the separate consideration of the half-cell reactions is on the heat distribution. This illustrates the significance of the transported ion entropy and how important it is to know this material-specific value.

10Sc1CeSZ is considered a promising electrolyte material for SOCs due to its stable cubic phase and high ionic conductivity [38,39]. Studies on the entropy change in the half-cell reactions, the transported ion entropy and the resulting information on the Peltier coefficient are not yet known. Therefore, the results of this work should close these data gaps. These data are determined from Seebeck coefficient measurements as a function of temperature, oxygen and hydrogen partial pressures. In addition, the anode-side Peltier heat is determined from the cathode-side Seebeck coefficient and the entropy change in the entire cell. The measurements are first carried out with an 8YSZ electrolyte to validate the procedure by comparing the results with the literature. Subsequently, the measurements are repeated with a 10SC1CeSZ electrolyte and compared with the previously obtained data.

## 2. Materials and Methods

### 2.1. Theory

**Figure 1:**
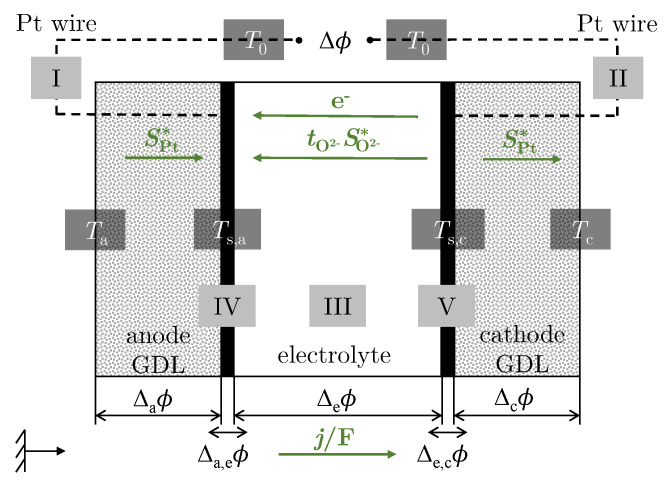
Schematic illustration of the oxygen concentration cell divided into the individual subsystems (I–V).

The following $O_{2}$ concentration cell is analysed:

(3) $$\mathrm{Pt}\left(T^{s,a}\right)|O_{2}\;\left(p^{s,a},T^{s,a}\right)||O^{2-}||O_{2}\;\left(p^{s,c},T^{s,c}\right)|\mathrm{Pt}\left(T^{s,c}\right),$$

with the anode-side electrochemical reaction equation:

(4) $$\begin{array}{cc} 1/2\;O^{2-} & \to 1/4\;O_{2}\;\left(g\right)+e^{-} \end{array}$$

and its back reaction at the cathode. The system is divided into five subsystems (I–V); see Figure 1. The EMF is measured between the Pt wires (I and II) at the hot ambient temperature $T_{0}$ in an open circuit. The temperatures at the contact points between the Pt wire and the cell housing correspond to the ambient temperature ($T_{a}=T_{c}=T_{0}$). The temperature of the anode (IV) is kept constant at $T_{s,a}$, while the temperature of the cathode (V) $T_{s,c}$ is changed so that a temperature difference ΔT = $T_{s,c}-T_{s,a}>0$ is set between the electrodes. The temperature difference ΔT leads to an electrical potential Δϕ, which is made up of the individual partial potentials. For small ΔT and j→0, the potential difference Δϕ is proportional to the temperature difference ΔT, with the resulting proportionality constant being the Seebeck coefficient according to [40]:

(5) $$\alpha_{S}={\left(\frac{d\phi }{dT}\right)}_{j\to 0,\;dT\to 0}=\frac{\Delta_{a}\phi +\Delta_{c}\phi }{dT}+\frac{\Delta_{e}\phi }{dT}+\frac{\Delta_{a,e}\phi +\Delta_{e,c}\phi }{dT}.$$

with $\Delta_{i}\phi$ as the potential difference in the homogeneous phase *i* (*a*: anode, *c*: cathode, *e*: electrolyte). Two indices are used to describe transport variables over the surface (s). The first subscript indicates the phase under consideration and the second subscript denotes the nearest phase. For example, $\Delta_{a,e}\phi$ is the difference in potential between the electrolyte (e) and the anode (a). The negative sign in the relationship between the Seebeck coefficient $\alpha_{S}$ and the Peltier coefficient π in Equation (2) results from the definition of Peltier heat. According to this definition, the reversible Peltier heat is absorbed at the anode when one Faraday positive charge is transported from the anode to the cathode. This also results in a positive definition of the electric current [40,41]. The transported entropy $S^{*}$ of the charge carriers follows from the entropic balance equation and $\pi =F\;{\left[J_{q}^{i}/j\right]}_{dT=0,d\mu =0}$ [42]:

(6) $$S^{*}=\frac{\pi }{T}=-F\cdot \alpha_{S}.$$

To establish the reversible entropy balance based on Equation (6) for the individual subsystems according to this definition, the green arrows shown in Figure 1 are used.

#### 2.1.1. Electric Leads

For the anode-side and cathode-side gas diffusion layer (GDL), oxygen is chosen as the reference frame so that $J_{O_{2}}=0$. The reversible entropy balances for the transport of electrons through the Pt wires are considered when one Faraday positive charge is transported from the anode to the cathode [41]:

(7) $$\begin{array}{cc} \Delta_{a}\phi & =-S_{\mathrm{Pt}}^{*}\left(T_{s,a}-T_{0}\right), \end{array}$$

(8) $$\begin{array}{cc} \Delta_{c}\phi & =-S_{\mathrm{Pt}}^{*}\left(T_{0}-T_{s,c}\right), \end{array}$$

so

(9) $$\frac{\Delta_{a}\phi +\Delta_{c}\phi }{dT}=S_{\mathrm{Pt}}^{*}\left(T\right).$$

With $S_{\mathrm{Pt}}^{*}$ as the transported entropy of the electrons in Pt, which can be determined from the data of Moore et al. [43]. *T* is the mean temperature of the both electrodes.

#### 2.1.2. Surfaces of the Electrodes

The Gibbs “Dividing Surface” approach is used to describe thermodynamic properties of surfaces between two homogeneous phases. The excess quantities introduced there serve to quantify the distribution and quantity of the respective property in comparison to the homogeneous phases. The approach, which was originally set up for a global equilibrium of the system, can be applied to the NET by taking into account time-dependent changes and defining a local equilibrium within a small volume element. For the complete anode-side, the excess entropy production rate ${\dot{\sigma }}^{s,a}$ is described according to this approach [40] by:

(10) $$\begin{array}{c} \;{\dot{\sigma }}^{s,a}=J_{q}^{a,e}\left(-\frac{1}{T^{a,e}}\frac{T^{s,a}-T^{a,e}}{T^{s,a}}\right)+J_{q}^{e,a}\left(-\frac{1}{T^{e,a}}\frac{T^{e,a}-T^{s,a}}{T^{s,a}}\right)+\cdots \\ \cdots +J_{O_{2}}^{e,a}\left[-\left(\frac{\mu_{O_{2}}^{s}}{T^{s,a}}-\frac{\mu_{O_{2}}^{e,a}}{T^{e,a}}\right)\right]+j\left[-\frac{1}{T^{s,a}}\left(\phi^{e,a}-\phi^{a,e}\right)\right]+r^{s,a}\left(-\frac{1}{T^{s,a}}\Delta_{n}G^{s,a}\right). \end{array}$$

In contrast to the fluxes, the excess entropy production rate is independent of the reference frame. According to [44], the surface reference frame in which the surface is at rest is therefore selected for the fluxes. The description of the indices can be taken from Figure 1. The first two terms of the equation represent the contributions to the heat flow. This is followed by the term for the chemical potential of the oxygen, which describes the outgoing mass flow. This is followed by the contribution of the electrical work of the surface. The last part of the equation represents the term for the reaction rate. Equation (10) can be simplified by the following assumptions, cf. [40]:

- constant temperatures due to the surfaces: $T^{a,e}=T^{e,a}=T^{s,a}$
- chemical equilibrium for the absorbed oxygen: $\mu_{O_{2}}^{a,e}\left(T^{s,a}\right)=\mu_{O_{2}}^{e,a}\left(T^{s,a}\right)=\mu_{O_{2}}^{s}\left(T^{s,a}\right)$
- constant current: $r^{s,a}=-j/F$.

If the same assumptions are also made for the surface on the cathode side, the electrical potential differences in the surfaces in the reversible case follow:

(11) $$\begin{array}{cc} \Delta_{a,e}\phi = & -\Delta_{n}G^{s,a}/F=-\frac{1}{F}\left(\frac{1}{4}\mu_{O_{2}}^{s,a}\left(T^{s,a}\right)\right), \end{array}$$

(12) $$\begin{array}{cc} \Delta_{e,c}\phi = & -\Delta_{n}G^{s,c}/F=-\frac{1}{F}\left(-\frac{1}{4}\mu_{O_{2}}^{s,c}\left(T^{s,c}\right)\right). \end{array}$$

The relationship ${\left(\delta \mu_{O_{2}}/\delta T\right)}_{p}=-S_{O_{2}}$ results:

(13) $$\frac{\Delta_{a,e}\phi +\Delta_{e,c}\phi }{dT}=-\frac{1}{F}\left(\frac{1}{4}S_{O_{2}}\left(T\right)\right).$$

The molar entropy $S_{m,k}$ of component *k* at temperature *T* is calculated by the entropic equation of state of ideal gases with the approach according to Kabelac et al. [45] for the molar isobaric heat capacities.

#### 2.1.3. Electrolyte

As in [3], the positive ion lattice is chosen as the homogeneous phase in the electrolyte as a reference frame so that $t_{O^{2-}}=1$ applies to the transport number of ions. When a positive charge of a Faraday passes from the anode to the cathode, the reversible entropy balance follows [41]:

(14) $$\frac{\Delta_{e}\phi }{dT}=-\frac{\pi_{e}}{T^{s,a}}=\frac{1}{2}S_{O^{2-}}^{*}\left(T^{s,a}\right).$$

$S_{O^{2-}}^{*}$ is the transported entropy of the ions. As already mentioned in the introduction, there are some studies on the transported entropy of the ions, which result in a determined value range of $S_{O^{2-}}^{*}=42-72\;J/\mathrm{KF}$ according to [33].

#### 2.1.4. Summary of the $O_{2}$ Concentration Cell

From the consideration of the individual subsystems, the Seebeck coefficient of the oxygen concentration cell can be calculated according to Equation (5). Thus, the Seebeck coefficient of the oxygen concentration cell results in:

(15) $$\alpha_{S,O_{2}}={\left(\frac{d\phi }{dT}\right)}_{j\to 0,\;dT\to 0}=\frac{1}{F}\left(-\frac{1}{4}S_{O_{2}}+S_{\mathrm{Pt}}^{*}+\frac{1}{2}S_{O^{2-}}^{*}\right),$$

from which the transported entropy of the ions $S_{O^{2-}}^{*}$ can be calculated by direct measurement of the Seebeck coefficient (as $S_{O_{2}}$ and $S_{\mathrm{Pt}}^{*}$ are known). Equation (14) can then be used to determine the Peltier coefficient of the electrolyte $\pi_{e}$. The Peltier coefficient for the oxygen reaction then follows from the relationship from Equation (6), cf. [3]:

(16) $$\frac{\pi_{c}}{T^{s,c}}=-\frac{1}{4}S_{O_{2}}+S_{\mathrm{Pt}}^{*}.$$

#### 2.1.5. Extension to $H_{2}/H_{2}O-O_{2}$ Cell

To determine the Peltier coefficient for the hydrogen reaction, a $H_{2}/H_{2}O$ concentration cell is now considered according to the following equation:

(17) $$\mathrm{Pt}\left(T^{s,a}\right)|H_{2}\;\left(p^{s,a},T^{s,a}\right),H_{2}O\;\left(p^{s,a},T^{s,a}\right)||O^{2-}||H_{2}\;\left(p^{s,c},T^{s,c}\right),H_{2}O\;\left(p^{s,a},T^{s,a}\right)|\mathrm{Pt}\left(T^{s,c}\right).$$

with the anode-side electrochemical reaction equation:

(18) $$\begin{array}{cc} 1/2\;H_{2}+1/2\;O^{2-} & \to e^{-}+1/2\;H_{2}O \end{array}$$

and its back reaction at the cathode. Analogous to the $O_{2}$ concentration cell, the same procedure can also be applied to the $H_{2}/H_{2}O$ concentration cell so that the Seebeck coefficient $\alpha_{S,H_{2}}$ applies:

(19) $$\alpha_{S,H_{2}}={\left(\frac{d\phi }{dT}\right)}_{j\to 0,\;dT\to 0}=\frac{1}{F}\left(-\frac{1}{2}S_{H_{2}O}+\frac{1}{2}S_{H_{2}}+S_{\mathrm{Pt}}^{*}+\frac{1}{2}S_{O^{2-}}^{*}\right),$$

so that the Peltier coefficient of the hydrogen reaction results according to [3]:

(20) $$\frac{\pi_{a}}{T^{s,a}}=-\frac{1}{2}S_{H_{2}O}+\frac{1}{2}S_{H_{2}}+S_{\mathrm{Pt}}^{*}.$$

From the divided consideration of the individual half-cell reactions, the entropy change ΔS or reaction entropy of the $H_{2}/H_{2}O-O_{2}$ reaction for the isothermal case [41] follows by combination:

(21) $$F\left(\frac{d\phi_{O_{2}}}{dT}\right)-F\left(\frac{d\phi_{H_{2}}}{dT}\right)=F\left(\frac{d\phi_{\mathrm{tot}}}{dT}\right)=\frac{1}{2}S_{H_{2}O}-\frac{1}{4}S_{O_{2}}-\frac{1}{2}S_{H_{2}}=\Delta S,$$

while $\alpha_{S,O_{2}}=\left(d\phi_{O_{2}}/dT\right)$ corresponds to the Seebeck coefficient in Equation (15). If the potential difference in the case of the oxygen concentration cell $\alpha_{S,O_{2}}=\left(d\phi_{O_{2}}/dT\right)$ and the potential difference in the $H_{2}/H_{2}O-O_{2}$ cell $\alpha_{S,\mathrm{tot}}=\left(d\phi_{\mathrm{tot}}/dT\right)$ are measured, the Seebeck coefficient $\alpha_{S,H_{2}}=\left(d\phi_{H_{2}}/dT\right)$ of an $H_{2}/H_{2}O$ concentration cell can be calculated according to Equation (21). A plausibility check of the measured Seebeck coefficient $d\phi_{\mathrm{tot}}/dT$ can be performed from the calculation of the total reaction entropy ΔS. In addition, the transported entropy of the ions can then be calculated using Equation (19) to determine the transported entropy of the ions and compare it with the calculated values from Equation (15), which must be identical.

### 2.2. Experiments

#### Test Environment

**Figure 2:**
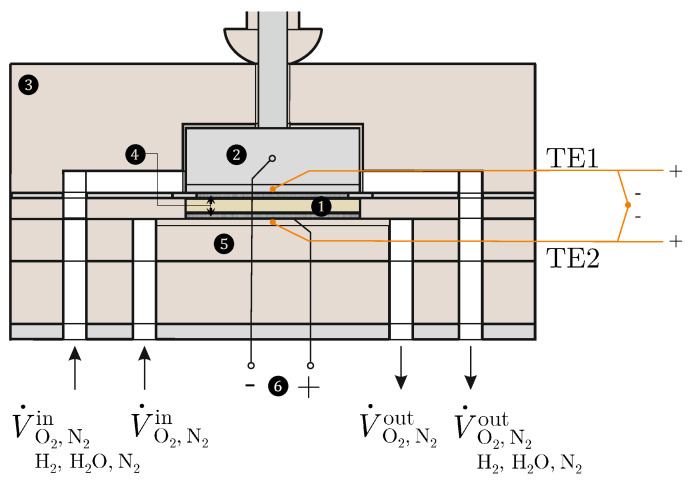
Measuring chamber: (1) sample, (2) inconel block with channels, (3) housing, (4) Pt mesh, (5) ceramic plate with channels and heater, (6) Pt wires.

The Seebeck coefficients according to Equations (15) and (21) are measured using two different test environments; see Figure 2.

For this purpose, an SOFC/SOEC test bench (Evaluator C1000-HT ) from HORIBA FuelCon (Magdeburg-Barleben, Germany) is available, which is suitable for analysing individual components. For the realisation of the oxygen concentration cell, only oxygen ($O_{2}$) and nitrogen ($N_{2}$) are available as gases for both electrodes, whereby different partial pressures of the oxygen can be set. In contrast, in the $H_{2}/H_{2}O-O_{2}$ cell, hydrogen ($H_{2}$), water ($H_{2}O$) and nitrogen ($N_{2}$) are supplied to the anode-side electrode. The volume flows of the gases can be precisely controlled in the range from 0 to 0.5 Nl/min using mass flow controllers. The test specimens (1) are 8YSZ and 10Sc1CeSZ oxide ceramic specimens with the dimensions 40 × 40 × 10 mm from CerPoTech (Heimdal, Norway). The surfaces of both test specimens are coated with a uniform 300 μm thick Pt coating by sputtering, which makes them electrochemically active. Pt nets (4) lie on these surfaces, which are in contact with Pt wires (6), via which the potential difference is measured. The Solartron ModuLab XM ECS electrochemical test system from AMETEK SI (Meerbusch, Germany) is available for this purpose. The ceramic base (5) contains flow channels for the cathode-side gases. A NiCr heating wire (Nikrothal^®^ 80) from Kanthal GmbH (Mörfelden-Walldorf, Germany), which is controlled by an external voltage source, is placed inside these channels. The heating wire is electrically insulated from the Pt network using a ceramic paste. The temperature difference $\Delta T=T^{s,c}-T^{s,a}>0$ is measured directly by connecting two type-S thermocouples (TE1 and TE2) with opposite poles. These thermocouples are attached to the top and bottom of the sample and are calibrated using the Pegasus 4853 high-temperature calibrator from ISOTECH (Merseyside, England). The test environment is closed off by an oven that is heated to a temperature of $\vartheta_{\mathrm{amb}}$ = 1000 °C. The manufacturers give the uncertainties of all measuring instruments used. The GUM [46] and DIN EN 60584-1 [47] are used to determine the measurement uncertainty and the limiting deviation.

## 3. Experimental Calculation

After heating the measuring chamber (mc), the gas tightness of the set-up was determined by comparing the measured open circuit voltage (OCV) and the calculated Nernst voltage for different partial pressure ratios $x_{O_{2}}=\ln\left(p_{O_{2}}^{c}/p_{O_{2}}^{a}\right)$, also $x_{H_{2}/H_{2}O-O_{2}}=\ln\left(p_{H_{2}O}/p_{H_{2}}\cdot p_{O_{2}}^{0.5}\right)$ was checked. This electrochemical leak test was carried out for measuring chamber temperatures of $\vartheta_{\mathrm{mc}}$ = 700 °C, 800 °C and 900 °C. To additionally check the influence of the total volume flow rates at the electrodes, the measurements were repeated once with ${\dot{V}}_{\mathrm{total}}=0.2\;\mathrm{Nl}/\min$ and ${\dot{V}}_{\mathrm{total}}=0.5\;\mathrm{Nl}/\min$. All measurements showed good agreement with the calculated Nernst voltage. During the operation of the oxygen concentration cell, maximum deviations of 15% were found for high partial pressure ratios of $x_{O_{2}}=\pm \;2.3$, which can be attributed to the limited accuracy of the mass flow control at low oxygen volume flows. In $H_{2}/H_{2}O-O_{2}$ operation, the maximum deviations were 1.5 %.

The gas tightness was rated as sufficient, so an impairment of the Pt coatings on the electrochemical reaction could be ruled out.

### 3.1. $O_{2}$ Cell

Three different experiments are carried out in the operation of the oxygen cell. To investigate the influence of temperature dependence, these experiments are repeated at temperatures $\vartheta_{\mathrm{mc}}=700\;{}^{\circ}C,\;800\;{}^{\circ}C$ and 900°C. The total volume flow rates are identical on both electrodes, ${\dot{V}}_{\mathrm{total}}^{a}={\dot{V}}_{\mathrm{total}}^{c}$. As described above, the surface temperature at the cathode $T^{c}$ is heated evenly by a heating wire. Temperature differences of ΔT=0−15K are approached. The three experiments are carried out as follows:

(1) Identical oxygen composition at both electrodes, $x_{O_{2}}^{a}=x_{O_{2}}^{c}=0.1,0.2,0.5,0.8,1$,
(2) Variation in the anode-side oxygen composition $x_{O_{2}}^{a}=0.1,0.2,0.5,0.8,1$ with a constant cathode-side oxygen composition of $x_{O_{2}}^{c}=1$,
(3) Variation in the cathode-side oxygen composition $x_{O_{2}}^{c}=0.1,0.2,0.5,0.8,1$ with a constant anode-side oxygen composition of $x_{O_{2}}^{a}=1$.

### 3.2. $H_{2}/H_{2}O-O_{2}$ Cell

Three different experiments are also carried out for this operation. The framework conditions with regard to the volume flows and the temperature difference correspond to those used in the operation of the oxygen concentration cell. The three experiments are carried out as follows:

(4) Variation in the cathode-side composition $x_{O_{2}}^{c}=0.1,0.21,0.4,1$ with an anode-side composition according to $x_{H_{2}}^{a}+x_{H_{2}O}^{a}=1$ at $\vartheta_{\mathrm{mc}}=700\;{}^{\circ}C$,
(5) Variation in the anode-side composition according to $x_{H_{2}}^{a}+x_{H_{2}O}^{a}+x_{N_{2}}^{a}=1$ with a cathode-side composition of $x_{O_{2}}^{c}=0.21$ for $\vartheta_{\mathrm{mc}}=700\;{}^{\circ}C$,
(6) Variation in the measuring chamber temperature $\vartheta_{\mathrm{mc}}=700\;{}^{\circ}C,800\;{}^{\circ}C,900\;{}^{\circ}C$ with an anode-side composition of $x_{H_{2}}^{a}+x_{H_{2}O}^{a}=1$ and a cathode-side composition of $x_{O_{2}}^{c}=0.21$.

## 4. Results and Discussion

### 4.1. $O_{2}$ Cell

#### 4.1.1. Seebeck Coefficient

**Figure 3:**
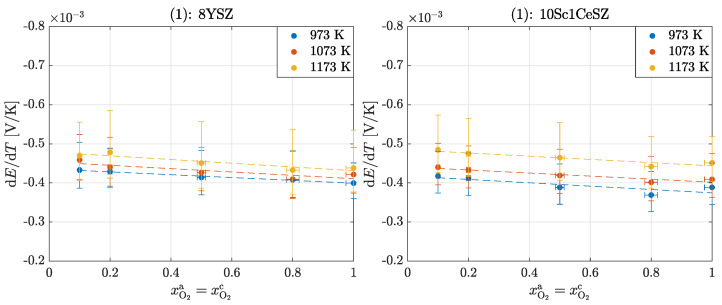
Seebeck coefficients $\alpha_{S,O_{2}}$ in the operation of an oxygen concentration cell for experiment (1): 8YSZ and 10Sc1CeSZ.

Figure 3 shows the measured Seebeck coefficients $\alpha_{S,O_{2}}=dE/dT$ for an oxygen concentration cell with identical oxygen composition at both electrodes for the temperatures $\vartheta_{\mathrm{mc}}=700\;{}^{\circ}C,800\;{}^{\circ}C$ and 900°C for 8YSZ and 10Sc1CeSZ.

The Seebeck coefficients of 8YSZ measured in this experiment are slightly below the values given in the literature; see Table 1. However, since there are also large scatters between the literature values and the measured values are also subject to some errors, the results are assessed as plausible. The large vertical error bars result from the temperature measurement at the temperatures under consideration. The horizontal error bars result from the uncertainties of the mass flow controllers. This should explain that there is no true value in the literature, but that rather all measured values can be considered valid. The negative signs indicate that heat is generated on the anode-side surface and is reversibly transported inside the cell from the cold surface to the hot surface.

The following applies to the dependence of the Seebeck coefficient on the oxygen partial pressure $\alpha_{S,O_{2}}\left(p_{O_{2}}\right)$:

(22) $$\left(\frac{\alpha_{S,O_{2}}}{d\ln p_{O_{2}}/p_{0}}\right)=\frac{R}{4F}=0.0215\;\mathrm{mV}/K,$$

in which the following dependencies can be determined from the available results; see Table 2.

Figure 3 shows a slight temperature dependence of the Seebeck coefficient $\alpha_{S,O_{2}}\left(T\right)$, according to [33]:

(23) $$\left(\frac{\alpha_{S,O_{2}}}{d\ln T/T_{0}}\right)=\frac{1}{F}\left(-\frac{1}{4}C_{m,p,O_{2}}+\tau_{\mathrm{Pt}}^{*}+\frac{1}{2}\tau_{O^{2-}}^{*}\right).$$

For 8YSZ, this results in a negative slope of $-0.00022\pm 0.0003\;\mathrm{mV}/K^{2}$ and for 10Sc1CeSZ of $-0.00031\pm 0.00026\;\mathrm{mV}/K^{2}$. There is no explicit temperature dependence addressed in the literature [32]. Some studies claim that a temperature dependence arises only from the molar isobaric heat capacity of oxygen $C_{m,p,O_{2}}$ and the Thomson coefficient of the electron $\tau_{\mathrm{Pt}}^{*}$ so that these two terms cancel each other out and there is no temperature dependence of the transported entropy of the oxygen ion results [48]. Other studies show temperature independence of the Seebeck coefficient, neglect the Thomson coefficient of the electron and equate the Thomson coefficient of the ion $\tau_{O^{2-}}^{*}$ with the molar isobaric heat capacity [33]. From all considerations, as well as from the results here, a very low temperature dependence (of $\alpha_{S,O_{2}}$ or $S_{O^{2-}}^{*}$) results. A comparison of the Seebeck coefficients of the two materials shows that the relative deviation of 10Sc1CeSZ to 8YSZ at 700°C is about −5.5%, at 800°C about −2.55% and at 900°C is even 2.02%. These values show that the Seebeck coefficient of 8YSZ is slightly higher than that of 10Sc1CeSZ at higher temperatures, with differences decreasing with increasing temperature. This correlation is established by the absolute measurement results. Due to the presence of large error bars, this statement is only qualitative. The studies by Ahlgreen et al. [28] and Pizzini et al. [31] show that the Seebeck coefficient decreases with increasing doping and increasing vacancies. However, the differences here are too small to give a valid conclusion.

**Figure 4:**
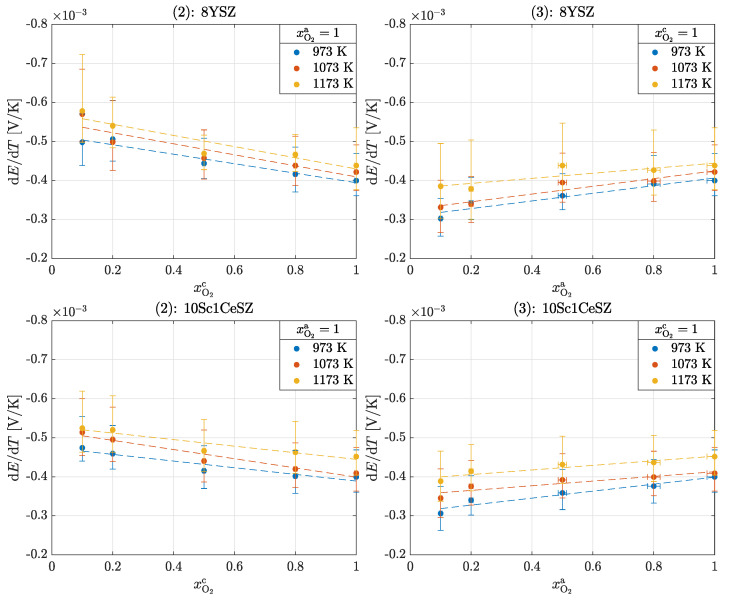
Seebeck coefficients $\alpha_{S,O_{2}}$ in the operation of an oxygen cell for experiments (2) and (3): 8YSZ and 10Sc1CeSZ.

Figure 4 shows the results of experiments (2) and (3) for 8YSZ and 10Sc1CeSZ. The fundamental differences in terms of temperature and material dependence can also be seen in these experiments, as in the previous experiment. A clear difference to experiment (1) can be seen in the slopes of the Seebeck coefficients, which are more pronounced in these experiments; see Table 3.

As in experiment (1), it can be seen in experiment (2) that the Seebeck coefficients increase with the ln of the decreasing oxygen partial pressure. This effect is further enhanced by the difference in composition. Equation (15) cannot be used for these experiments because there are different oxygen partial pressures at the electrodes; see Equation (12). If experiment (2) is reversed, the results from experiment (3) are obtained. The behavior of the Seebeck coefficients is reversed, so they decrease with the ln of the decreasing oxygen partial pressure.

#### 4.1.2. Transported Ion Entropy and Peltier Coefficient

The transported entropy of the oxygen ions $S_{O^{2-}}^{*}$ and the Peltier coefficients of the cathode $\pi_{c}$ and the electrolyte $\pi_{e}$ are calculated from the results of experiment (1) using Equations (14) and (16). In contrast to the Peltier coefficient of the cathode, the mean values $\hat{S_{O^{2-}}^{*}}$ and $\hat{\pi_{e}}$ are calculated due to the independence of the oxygen partial pressure. Measurement inaccuracies are taken into account when calculating the mean values. For reasons of clarity, the uncertainties of all individual values have been omitted. The results are summarized in Table 4.

Due to the direct dependence of the transported entropy of the ions $S_{O^{2-}}^{*}\left(\alpha_{S,O_{2}}\right)$ on the Seebeck coefficient, this shows a corresponding behavior with changes in the oxygen partial pressure, the temperature and the material. The values determined are slightly above the previously assumed value of $S_{O^{2-}}^{*}$ = 42 J/KF at 1273 K according to [33]. This also results in slightly higher Peltier coefficients for the electrolyte $\pi_{e}\left(S_{O^{2-}}^{*}\right)$. The influence of the transported entropy of the ions is examined in more detail in the last part of the results. If the Peltier coefficients of the two homogeneous phases are compared, it can also be stated here that the effect is more pronounced at the cathode than in the electrolyte, which has already been shown in the modeling results [3,4].

### 4.2. $H_{2}/H_{2}O-O_{2}$ Cell

**Figure 5:**
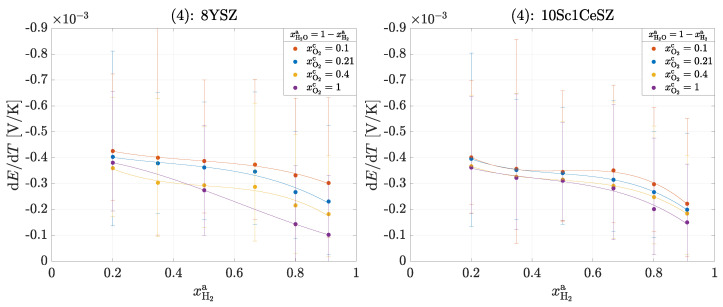
Seebeck coefficients $\alpha_{S,\mathrm{tot}}$ in the operation of a $H_{2}/H_{2}O-O_{2}$ cell for experiment (4): 8YSZ and 10Sc1CeSZ. The solid lines within a measurement series are used for clear visualization.

Figure 5 shows the measured Seebeck coefficients $\alpha_{S,\mathrm{tot}}=dE/dT$ for an $H_{2}/H_{2}O-O_{2}$ cell for experiment (4). With the help of the following results, the transported entropy of the ions $S_{O^{2-}}^{*}$ can also be determined; these must then correspond to the values from Table 4. Furthermore, the Peltier coefficient of the homogeneous phase on the anode side $\pi_{a}$ is calculated according to Equation (20).

**Figure 6:**
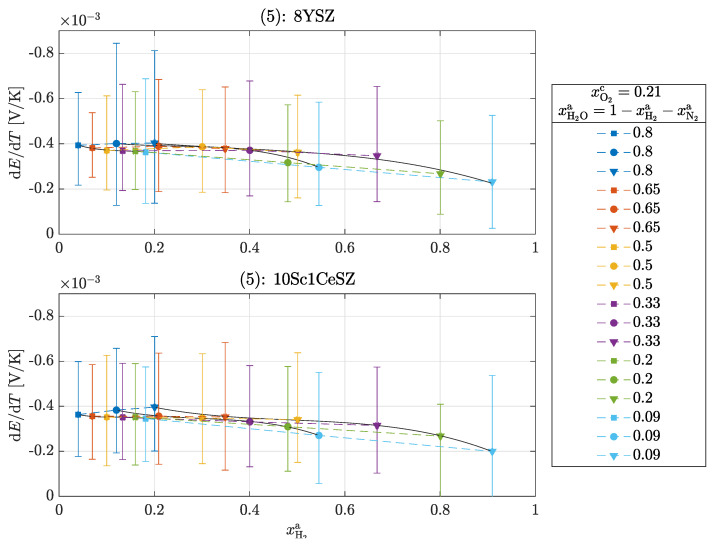
Seebeck coefficients $\alpha_{S,\mathrm{tot}}$ in the operation of a $H_{2}/H_{2}O-O_{2}$ cell for experiment (5): 8YSZ and 10Sc1CeSZ. The solid lines within a measurement series are used for clear visualization.

According to Equation (21), the overall reaction is exothermic at every operating point ($\alpha_{S,\mathrm{tot}}<0$). It can be seen that with increasing oxygen partial pressure at the cathode and with increasing hydrogen partial pressure at the anode, the Seebeck coefficient decreases and thus less heat is released. There is no clear difference between the materials, which is to be expected and seems to be correct, as the same reaction entropy ΔS is released in both materials. It is remarkable that the order of magnitude of the Seebeck coefficients here is similar to that in Figure 3. At an oxygen partial pressure of $p_{O_{2}}=0.21$ bar, experiment (1) results in a value of −0.43 ± 0.05 mV/K for 8YSZ and here with increasing hydrogen partial pressure $p_{H_{2}}$ from −0.4 ± 0.3 to −0.2 ± 0.2 mV/K. From this observation, it can be concluded that heat production on the cathode-side surface dominates the overall reaction. The following Figure 6 shows the measured Seebeck coefficients $\alpha_{S,\mathrm{tot}}=dE/dT$ for experiment (5).

**Figure 7:**
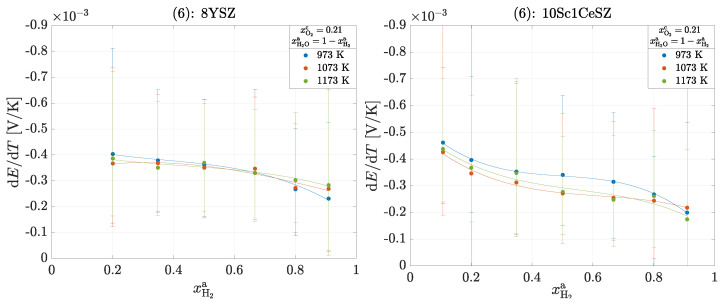
Seebeck coefficients $\alpha_{S,\mathrm{tot}}$ in the operation of a $H_{2}/H_{2}O-O_{2}$ cell for experiment (6): 8YSZ and 10Sc1CeSZ. The solid lines within a measurement series are used for clear visualization.

Operating points of the same color mean that the supplied volume flow of water ${\dot{V}}_{H_{2}O}$ was kept constant and the volume flows of hydrogen and nitrogen were varied so that ${\dot{V}}_{\mathrm{total}}$ = const. This resulted in a constant water partial pressure $p_{H_{2}O}$ with a varrying $p_{H_{2}}/p_{N_{2}}$ ratio. Continuous operating points typically have a constant $p_{H_{2}}/p_{N_{2}}$ ratio with varying water partial pressure $p_{H_{2}O}$. Here, too, the Seebeck coefficients decrease with increasing hydrogen partial pressure. However, the Seebeck coefficients show deviations from this behavior within a constant water partial pressure; here, $p_{H_{2}O}=0.8$ and $p_{H_{2}O}=0.65$. With increasing hydrogen partial pressure, slight increases can be seen in these points, which does not correspond to theory. One possible reason for the deviation is the inaccurate control of the hydrogen supply quantity at these low partial pressures. Finally, the results of experiment (6) are shown in Figure 7.

As in the operation of the oxygen concentration cell, the temperature dependence should be determined here. There are no clear dependencies for either material. According to theory, the following applies to an electron $F\cdot \Delta^{R}S_{m}\left(700\;{}^{\circ}C,x_{H_{2}O}=x_{H_{2}}=0.5,\;x_{O_{2}}=0.21\right)=0.318\;V/K$ and $F\cdot \Delta^{R}S_{m}\left(900\;{}^{\circ}C,x_{H_{2}O}=x_{H_{2}}=0.5,x_{O_{2}}=0.21\right)=0.324\;V/K$ for 8YSZ. Accordingly, the total reaction entropy $\Delta^{R}S_{m}$ and thus also the Seebeck coefficient increase with increasing temperature. However, this change is so small that it is not clearly seen within the measurement inaccuracies.

#### 4.2.1. Transported Ion Entropy and Peltier Coefficient

Table 5 summarizes the transported entropies of the ions $S_{O^{2-}}^{*}$, the Peltier coefficients of the electrolyte $\pi_{e}$ and the anode $\pi_{a}$ from the results of Figure 5 according to Equations (19), (20) and (14). First, the mean values for $S_{O^{2-}}^{*}$ and $\pi_{e}$ were calculated at $p_{O_{2}}=\mathrm{const}.$ and then the total mean value was calculated from these individual mean values. These values are compared with the values from Table 4 and it can be seen that the values match with a slight inaccuracy, which corresponds to the theory. It can also be seen that the Peltier coefficients of the homogeneous phase on the anode side are about half as large as the Peltier coefficients on the cathode side, which illustrates the stronger manifestation of the Peltier effect at the cathode.

Experiment (5) also shows very good agreement with the results from the oxygen concentration cell; see Table 6. A closer look at the transported ion entropy for the two materials shows higher values for 8YSZ than for 10Sc1CeSZ in $H_{2}/H_{2}O-O_{2}$ mode. However, based on the previously obtained results and the findings from the literature, this behavior should be reversed. The deviations are due to the measurement in accuracies. The same behavior can also be seen in the results of experiment (6); see Table 7. Furthermore, a clear temperature dependence of the transported ion entropy can also be seen here. The significance of the results obtained for the operational behavior of an SOFC/SOEC is discussed below.

#### 4.2.2. Heat Generation of the Electrodes of an SOC

**Figure 8:**
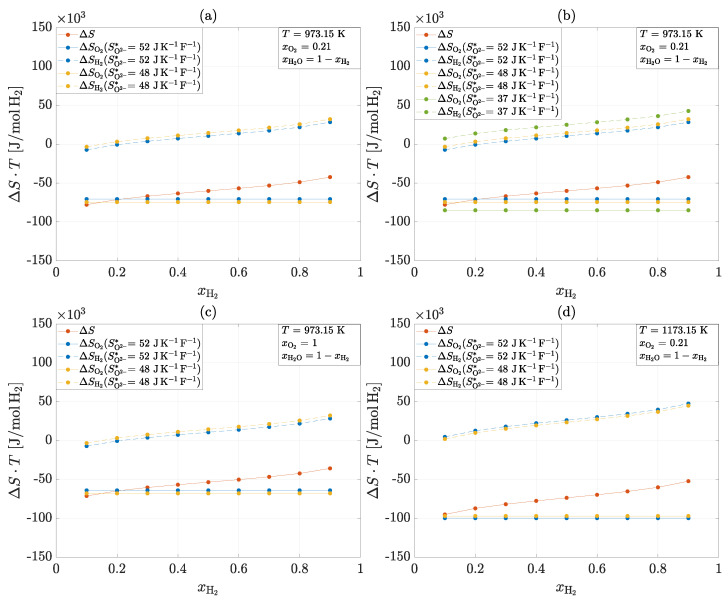
Total heat production and the partial heat production at the electrodes for four different scenarios: 1. Reference scenario (**a**). 2. Variation in the transported entropy of the ion (**b**). 3. Change in oxygen patial pressure from $p_{O_{2}}=0.21$ bar to $p_{O_{2}}=1$ bar (**c**). 4. Change in temperature from T=973.15 K to T=1173.15 K (**d**).

The results for the transported entropy of the ions $S_{O^{2-}}^{*}$ from experiments (1), (4), (5) and (6) can be used to calculate the heat generation of the half-cell reactions of an SOC. Figure 8 illustrates the total heat production T·ΔS and the partial heat production at the electrodes $T\cdot \Delta S_{i}$ for four different scenarios based on the results obtained.

The overall reaction in all scenarios is exothermic and consists of an exothermic reaction at the cathode and a predominantly endothermic reaction at the anode. The reference scenario (a) describes an operation at a temperature of T=973.15 K and a constant oxygen partial pressure on the cathode side of $p_{O_{2}}=\mathrm{const}.=0.21$ bar. The transported ion entropy is $S_{O^{2-}}^{*}=52\pm 10$ J/KF for 10Sc1CeSZ and $S_{O^{2-}}^{*}=48\pm 9$ J/KF for 8YSZ. It can be seen that there are minimal differences in the partial heat production of the individual electrodes between the two materials. In general, it can be seen that 10Sc1CeSZ produces slightly less heat at the cathode and absorbs slightly less heat at the anode than 8YSZ.

Taking the same operating conditions, the influence of the previously assumed transported ion entropy of $S_{O^{2-}}^{*}=37$ J/KF is also determined in addition to the reference scenario (b). As this value is significantly lower, this results in higher overall amounts of heat that are absorbed or released at the electrodes. It can also be seen that an endothermic anode reaction occurs at low hydrogen partial pressures. This finding is also shown by Fischer et al. [37].

If oxygen is supplied instead of air on the cathode side, only slight differences in the total heat production and the heat production on the cathode side are recognizable (c). Increasing the oxygen partial pressure leads to less heat being released at the cathode, which results in a slight reduction in total heat production.

The last scenario examined is the influence of temperature (d). An increase in temperature by T=200 K leads to a significant increase in heat production in the overall reaction and at the cathode. Although more heat is also absorbed in the anode, the exothermic part of the cathode reaction dominates.

## 5. Conclusions

For exact modeling and calculation of the heat transport in SOCs, the transported entropy of the ions for an 8YSZ and a 10Sc1CeSZ electrolyte was determined experimentally in this work, from which, in turn, the Peltier coefficient could be calculated directly. The Peltier effect leads to the transport of reversible heat in the cell. In our previous studies and also in the literature, it has already been clearly shown that this effect must be taken into account for successful thermal management. In contrast to 10Sc1CeSZ, some data exist for 8YSZ in the literature, so the plausibility of these results could be shown. The transported entropy of the ions was determined by direct measurement of the Seebeck coefficient. This was measured for a $O_{2}$ concentration cell, a $O_{2}-O_{2}$ cell and a $H_{2}/H_{2}O-O_{2}$ cell in the temperature range from 700 to 900°C. The different operating modes were used for methodological variation but should provide and do provide consistent results.

The results from the $O_{2}$ concentration cell for 8YSZ show good agreement with the results from the literature. It was found that the measured Seebeck coefficients of 10Sc1CeSZ are slightly smaller than those of 8YSZ. This results in a higher transported entropy of the ions. For a temperature of 700°C and an oxygen partial pressure of $p_{O_{2}}=0.21$ bar in a value of $S_{O^{2-}}^{*}=52\pm 10$ J/KF for 10Sc1CeSZ and $S_{O^{2-}}^{*}=48\pm 9$ J/KF for 8YSZ results. The determination of the transported entropy of the ions via the operation of a $H_{2}/H_{2}O-O_{2}$ cell has not been shown previously in the literature. The validity of the measured Seebeck coefficients could be demonstrated with a theoretical calculation of the total reaction entropy. The results for the transported entropy of the ions showed deviations of about 6% at 8YSZ and −2.6% at 10Sc1CeSZ compared to the results from the $O_{2}$ concentration cell operation. However, taking into account the measurement errors, these deviations were assessed as small. The influence of the transported entropy of the ions, the oxygen partial pressure and the temperature on the total heat generation and on the partial heat generation of the electrodes of a $H_{2}/H_{2}O-O_{2}$ cell was also investigated. As expected, the temperature has the greatest influence on the heat generation of the half-cell reactions and thus also on the total heat generation in the cell. The transported entropy of the ions has an effect of $T\cdot S_{O^{2-}}^{*}$ on the heat generation of both half-cell reactions.

Based on the successful measurement results, values for the Peltier coefficients were determined of the homogeneous phases (anode, cathode and electrolyte) that correspond to those in the literature. A negative sign of the coefficient was found for all phases, whereby the coefficient for the homogeneous phase of the cathode was twice as high as that for the anode and the electrolyte. Previous studies have already shown that the Peltier effect at the cathode has the greatest influence on the system.

## Figures and Tables

**Table 1:** Seebeck coefficients for $Y_{2}O_{3}$ and ${\mathrm{Sc}}_{2}O_{3}$ ceramics from the literature.

T [K]	Dopant	mol%	$p_{O_{2}}$ [bar]	$-\alpha_{S}$ [mV/K]	Reference
960–1310	$Y_{2}O_{3}$	9	$-\alpha_{S}=0.46+0.0219\cdot \ln\left(159/\left(p_{O_{2}}\cdot 750\right)\right)$		[29]
1448	$Y_{2}O_{3}$	10	0.21	0.47	from [28]
		20		0.44	
		40		0.42	
1873	$Y_{2}O_{3}$	4.5	1	0.503	from [28]
		5.8		0.498	
		6.8		0.486	
		7.6		0.485	
		8.4		0.471	
1273	$Y_{2}O_{3}$	8	0.21	0.303	[34]
1186	$Y_{2}O_{3}$	8	0.21	0.46	[35]
1273	$Y_{2}O_{3}$	8	0.21	0.423	[36]
1186	$Y_{2}O_{3}$	8	0.21	0.43	[32]
			1	0.46	
1273	$Y_{2}O_{3}$	3	0.21	0.498	[33]
		5		0.487	
		8		0.495	
		12		0.478	
1273	$Y_{2}O_{3}$	2	0.21	0.533	[28]
		8		0.486	
		18		0.405	
1273	${\mathrm{Sc}}_{2}O_{3}$	12.5	1	0.4	[28]
1873	${\mathrm{Sc}}_{2}O_{3}$	4.4	1	0.468	[28]
		6.9		0.467	
		8		0.461	
		8.7		0.46	

**Table 2:** Slope of the linear relationship between the Seebeck coefficient $\alpha_{S,O_{2}}$ and the partial pressure of oxygen $p_{O_{2}}$ for 8YSZ and 10Sc1CeSZ for $\vartheta_{\mathrm{mc}}=700\;{}^{\circ}C,800\;{}^{\circ}C,900\;{}^{\circ}C$; see Figure 3.

T [K]	Dopant	Slope [mV/K]
973.15	$Y_{2}O_{3}$	0.014 ± 0.022
973.15	${\mathrm{Sc}}_{2}O_{3}$	0.018 ± 0.017
1073.15	$Y_{2}O_{3}$	0.019 ± 0.023
1073.15	${\mathrm{Sc}}_{2}O_{3}$	0.016 ± 0.010
1173.15	$Y_{2}O_{3}$	0.018 ± 0.029
1173.15	${\mathrm{Sc}}_{2}O_{3}$	0.017 ± 0.014

**Table 3:** Slope of the linear relationship between the Seebeck coefficient $\alpha_{S,O_{2}}$ and the partial pressure of oxygen $p_{O_{2}}^{i}$ for 8YSZ and 10Sc1CeSZ for $\vartheta_{\mathrm{mc}}=700\;{}^{\circ}C,800\;{}^{\circ}C,900\;{}^{\circ}C$; see Figure 4.

T [K]	Dopant	Slope for $x_{O_{2}}^{a}=1$ [mV/K]	Slope for $x_{O_{2}}^{c}=1$ [mV/K]
973.15	$Y_{2}O_{3}$	0.047 ± 0.022	−0.04 ± 0.02
973.15	${\mathrm{Sc}}_{2}O_{3}$	0.035 ± 0.021	−0.036 ± 0.019
1073.15	$Y_{2}O_{3}$	0.06 ± 0.029	−0.04 ± 0.027
1073.15	${\mathrm{Sc}}_{2}O_{3}$	0.048 ± 0.026	−0.025 ± 0.023
1173.15	$Y_{2}O_{3}$	0.06 ± 0.04	−0.027 ± 0.034
1173.15	${\mathrm{Sc}}_{2}O_{3}$	0.034 ± 0.027	−0.024 ± 0.023

**Table 4:** Transported entropy of the ions $S_{O^{2-}}^{*}$ and Peltier coefficient for the electrolyte $\pi_{e}$ and cathode $\pi_{c}$ from the results in Figure 3.

T [K]	Dopant	$p_{O_{2}}$ [bar]	$S_{O^{2-}}^{*}$ [J/KF]	$\pi_{e}$ [J/C]	$\pi_{c}$ [J/C]
973.15	$Y_{2}O_{3}$	0.1	50	−0.25	−0.66
		0.21	48	−0.24	−0.65
		0.5	47	−0.24	−0.63
		0.8	47	−0.23	−0.62
		1	47	−0.23	−0.62
			$\hat{S_{O^{2-}}^{*}}=48\pm 9$	$\hat{\pi_{e}}=-0.24\pm 0.04$	
1073.15		0.1	47	−0.26	−0.74
		0.21	48	−0.26	−0.73
		0.5	47	−0.26	−0.71
		0.8	48	−0.26	−0.70
		1	45	−0.25	−0.69
			$\hat{S_{O^{2-}}^{*}}=47\pm 11$	$\hat{\pi_{e}}=-0.26\pm 0.06$	
1173.15		0.1	47	−0.28	−0.83
		0.21	43	−0.26	−0.81
		0.5	44	−0.26	−0.79
		0.8	46	−0.27	−0.77
		1	44	−0.26	−0.77
			$\hat{S_{O^{2-}}^{*}}=45\pm 14$	$\hat{\pi_{e}}=-0.27\pm 0.09$	
973.15	${\mathrm{Sc}}_{2}O_{3}$	0.1	53	−0.26	−0.66
		0.21	51	−0.25	−0.65
		0.5	52	−0.26	−0.63
		0.8	54	−0.27	−0.62
		1	49	−0.24	−0.62
			$\hat{S_{O^{2-}}^{*}}=52\pm 10$	$\hat{\pi_{e}}=-0.26\pm 0.05$	
1073.15		0.1	51	−0.26	−0.74
		0.21	49	−0.26	−0.73
		0.5	48	−0.26	−0.71
		0.8	49	−0.26	−0.70
		1	47	−0.25	−0.69
			$\hat{S_{O^{2-}}^{*}}=49\pm 10$	$\hat{\pi_{e}}=$ −0.27±0.06	
1173.15		0.1	44	−0.28	−0.83
		0.21	43	−0.26	−0.81
		0.5	41	−0.26	−0.79
		0.8	43	−0.27	−0.77
		1	40	−0.26	−0.77
			$\hat{S_{O^{2-}}^{*}}=42\pm 11$	$\hat{\pi_{e}}=-0.25\pm 0.07$	

**Table 5:** Transported entropy of the ions $S_{O^{2-}}^{*}$ and Peltier coefficient for the electrolyte $\pi_{e}$ and anode $\pi_{a}$ for T=973 K from the results in Figure 5.

Dopant	$p_{H_{2}}$ [bar]	$p_{O_{2}}$ [bar]	$S_{O^{2-}}^{*}$ [J/KF]	$\pi_{e}$ [J/C]	$\pi_{a}$ [J/C]
$Y_{2}O_{3}$	0.2	0.21	53	−0.26	−0.28
	0.35		54	−0.27	−0.32
	0.5		56	−0.28	−0.34
	0.67		59	−0.29	−0.37
	0.8		50	−0.25	−0.4
	0.91		50	−0.25	−0.44
			$\hat{S_{O^{2-}}^{*}}=54\pm 37$	$\hat{\pi_{e}}=-0.27\pm 0.2$	
$Y_{2}O_{3}$	0.2	1	54	−0.27	−0.28
	0.35		55	−0.27	−0.32
	0.5		45	−0.22	−0.34
	0.67		45	−0.22	−0.37
	0.8		31	−0.16	−0.4
	0.91		31	−0.16	−0.44
			$\hat{S_{O^{2-}}^{*}}=44\pm 39$	$\hat{\pi_{e}}=-0.22\pm 0.2$	
$Y_{2}O_{3}$	"	0.1	$\hat{S_{O^{2-}}^{*}}=60\pm 47$	$\hat{\pi_{e}}=-0.3\pm 0.2$	
$Y_{2}O_{3}$	"	0.4	$\hat{S_{O^{2-}}^{*}}=46\pm 38$	$\hat{\pi_{e}}=-0.23\pm 0.2$	
			$\hat{S_{O^{2-}}^{*}}=51\pm 40$	$\hat{\pi_{e}}=-0.25\pm 0.2$	
					
Table 4			$\hat{S_{O^{2-}}^{*}}=48\pm 9$	$\hat{\pi_{e}}=-0.24\pm 0.043$	
${\mathrm{Sc}}_{2}O_{3}$	0.2	0.21	51	−0.26	−0.28
	0.35		49	−0.25	−0.32
	0.5		52	−0.26	−0.34
	0.67		53	−0.26	−0.37
	0.8		50	−0.25	−0.4
	0.91		44	−0.22	−0.44
			$\hat{S_{O^{2-}}^{*}}=50\pm 38$	$\hat{\pi_{e}}=-0.25\pm 0.2$	
${\mathrm{Sc}}_{2}O_{3}$	0.2	1	53	−0.26	−0.28
	0.35		51	−0.25	−0.32
	0.5		54	−0.27	−0.34
	0.67		55	−0.27	−0.37
	0.8		52	−0.26	−0.4
	0.91		48	−0.24	−0.44
			$\hat{S_{O^{2-}}^{*}}=52\pm 36$	$\hat{\pi_{e}}=-0.26\pm 0.2$	
${\mathrm{Sc}}_{2}O_{3}$	"	0.1	$\hat{S_{O^{2-}}^{*}}=50\pm 40$	$\hat{\pi_{e}}=-0.25\pm 0.2$	
${\mathrm{Sc}}_{2}O_{3}$	"	0.4	$\hat{S_{O^{2-}}^{*}}=50\pm 36$	$\hat{\pi_{e}}=-0.25\pm 0.2$	
			$\hat{S_{O^{2-}}^{*}}=51\pm 38$	$\hat{\pi_{e}}=-0.25\pm 0.2$	
					
Table 4			$\hat{S_{O^{2-}}^{*}}=52\pm 10$	$\hat{\pi_{e}}=-0.26\pm 0.050$	

**Table 6:** Transported entropy of the ions $S_{O^{2-}}^{*}$ and Peltier coefficient for the electrolyte $\pi_{e}$ from the results in Figure 6.

T [K]	Dopant	$p_{H_{2}O}$ [bar]	$p_{H_{2}}$ [bar]	$\hat{S_{O^{2-}}^{*}}$ [J/KF]	$\hat{\pi_{e}}$ [J/C]
973.15	$Y_{2}O_{3}$	0.8	0.04, 0.12, 0.2	46	−0.23
		0.65	0.07, 0.21, 0.35	49	−0.25
		0.5	0.1, 0.3, 0.5	53	−0.26
		0.33	0.13, 0.4, 0.67	56	−0.28
		0.2	0.16, 0.48, 0.8	53	−0.26
		0.09	0.18, 0.55, 0.91	57	−0.28
				$\hat{S_{O^{2-}}^{*}}=52\pm 40$	$\hat{\pi_{e}}=-0.26\pm 0.2$
					
Table 4				$\hat{S_{O^{2-}}^{*}}=48\pm 9$	$\hat{\pi_{e}}=-0.24\pm 0.043$
973.15	${\mathrm{Sc}}_{2}O_{3}$	0.8	0.04, 0.12, 0.2	46	−0.23
		0.65	0.07, 0.21, 0.35	47	−0.23
		0.5	0.1, 0.3, 0.5	51	−0.25
		0.33	0.13, 0.4, 0.67	54	−0.27
		0.2	0.16, 0.48, 0.8	55	−0.27
		0.09	0.18, 0.55, 0.91	56	−0.28
				$\hat{S_{O^{2-}}^{*}}=51\pm 41$	$\hat{\pi_{e}}=-0.25\pm 0.2$
					
Table 4				$\hat{S_{O^{2-}}^{*}}=52\pm 10$	$\hat{\pi_{e}}=-0.26\pm 0.05$

**Table 7:** Transported entropy of the ions $S_{O^{2-}}^{*}$ and Peltier coefficient for the electrolyte $\pi_{e}$ from the results in Figure 7.

T [K]	Dopant	$\hat{S_{O^{2-}}^{*}}$ [J/KF]	$\hat{\pi_{e}}$ [J/C]
1073.15	$Y_{2}O_{3}$	51 ± 40	−0.27 ± 0.2
Table 4		47 ± 11	−0.26 ± 0.06
			
1173.15		48 ± 40	−0.29 ± 0.2
Table 4		44 ± 14	−0.27 ± 0.09
1073.15	${\mathrm{Sc}}_{2}O_{3}$	47 ± 40	−0.26 ± 0.2
Table 4		49 ± 10	−0.27 ± 0.06
			
1173.15		39 ± 39	−0.23 ± 0.2
Table 4		42 ± 11	−0.25 ± 0.07

## Data Availability

The original contributions presented in the study are included in the article, further inquiries can be directed to the corresponding author.

## Abbreviations

The following abbreviations are used in this manuscript:

8YSZ	8 mol% yttria stabilized zirconia
10Sc1CeSZ	10 mol% scandia and 1 mol% ceria stabilized zirconia
EMF	Electromotive force
GDL	Gas diffusion layer
NET	Non-equilibrium thermodynamics
OCV	Open circuit voltage
SOEC	Solid oxide fuel cell
SOFC	Solid oxide electrolyzer cell

## References

[1] Zeng Z., Qian Y., Zhang Y., Hao C., Dan D., Zhuge W. (2020). A review of heat transfer and thermal management methods for temperature gradient reduction in solid oxide fuel cell (SOFC) stacks. Appl. Energy.

[2] Kjelstrup Ratkje S., Bedeaux D., Johannessen E., Gross J. (2017). Non-Equilibrium Thermodynamics for Engineers.

[3] Gedik A., Lubos N., Kabelac S. (2022). Coupled Transport Effects in Solid Oxide Fuel Cell Modeling. Entropy.

[4] Gedik A., Wachtel J., Kabelac S. (2024). Comparative Analysis of Loss Mechanism Localization in a Semi-2D SOEC Single Cell Modell: Non-Equilibrium Thermodynamics versus Monocausal-Based Approach. ECS Adv..

[5] Børset M.T., Kang X., Burheim O.S., Haarberg G.M., Xu Q., Kjelstrup S. (2015). Seebeck coefficients of cells with lithium carbonate and gas electrodes. Electrochim. Acta.

[6] Kang X., Børset M.T., Burheim O.S., Haarberg G.M., Xu Q., Kjelstrup S. (2015). Seebeck coefficients of cells with molten carbonates relevant for the metallurgical industry. Electrochim. Acta.

[7] Barragán V.M., Kristiansen K.R., Kjelstrup S. (2018). Perspectives on Thermoelectric Energy Conversion in Ion-Exchange Membranes. Entropy.

[8] Kristiansen K.R., Barragán V.M., Kjelstrup S. (2019). Thermoelectric Power of Ion Exchange Membrane Cells Relevant to Reverse Electrodialysis Plants. Phys. Rev. Appl..

[9] Kjelstrup S., Kristiansen K.R., Gunnarshaug A.F., Bedeaux D. (2023). Seebeck, Peltier, and Soret effects: On different formalisms for transport equations in thermogalvanic cells. J. Chem. Phys..

[10] Gunnarshaug A.F., Vie P.J.S., Kjelstrup S. (2021). Review—Reversible Heat Effects in Cells Relevant for Lithium-Ion Batteries. J. Electrochem. Soc..

[11] Kamata M., Ito Y., Oishi J. (1987). Single electrode peltier heat of a hydrogen electrode in H2SO4 and NaOH solutions. Electrochim. Acta.

[12] Grimstvedt A., Ratkje S.K., Forland T. (1994). Theory of Thermocells: Transported Entropies, and Heat of Transfer in Sulfate Mixtures. J. Electrochem. Soc..

[13] Ratkje S.K., Ottøy M., Halseid R., Strømgård M. (1995). Thermoelectric power relevant for the solid-polymer-electrolyte fuel cell. J. Membr. Sci..

[14] Blinov V., Kjelstrup S., Bedeaux D., Sharivker V. (2001). The Role of the Transported Entropy of Lead Ions in Partially Thermostated and Adiabatic Cells. J. Electrochem. Soc..

[15] Fang Z., Wang S., Zhang Z., Qiu G. (2008). The electrochemical Peltier heat of the standard hydrogen electrode reaction. Thermochim. Acta.

[16] Kjelstrup S., Vie P., Akyalcin L., Zefaniya P., Pharoah J.G., Burheim O.S. (2013). The Seebeck coefficient and the Peltier effect in a polymer electrolyte membrane cell with two hydrogen electrodes. Electrochim. Acta.

[17] Gunnarshaug A.F., Kjelstrup S., Bedeaux D. (2020). The heat of transfer and the Peltier coefficient of electrolytes. Chem. Phys. Lett..

[18] Kiukkola K., Wagner C. (1957). Measurements on Galvanic Cells Involving Solid Electrolytes. J. Electrochem. Soc..

[19] Schmalzried H. (1962). Über Zirkondioxyd als Elektrolyt für elektrochemische Untersuchungen bei höheren Temperaturen. Z. Für Elektrochem. Berichte Der Bunsenges. Für Phys. Chem..

[20] Bray D.T., Merten U. (1964). Transport Numbers in Stabilized Zirconia. J. Electrochem. Soc..

[21] Steele B.C.H., Alcock C.B. (1965). Factors influencing the performance of solid oxide electrolytes in high temperature thermodynamic measurements. Trans. Met. Soc. AIME.

[22] Baker R., West J.M. (1966). Solid electrolytes for use at steelmaking temperatures. J. Iron Steel Inst..

[23] Fischer W.A., Janke D. (1972). Die Anwendbarkeit von ZrO2-Y2O3-, ZrO2-CaO- und ThO2-Y2O3-Festelektrolyten bei 1000 bis 1600 °C.

[24] Fischer W.A., Pieper C. (1973). Die elektrische Leitfähigkeit und Thermokraft von reinem und mit Calciumoxid stabilisiertem Zirkonoxid bei Temperaturen zwischen 1000 und 1700 °C und Sauerstoffpartialdrücken zwischen 1 und 10–16 atm. Arch. Für Das Eisenhüttenwesen.

[25] Park J.H., Blumenthal R.N. (1989). Electronic Transport in 8 Mole Percent Y_2_O_3_–ZrO_2_. J. Electrochem. Soc..

[26] Kosacki I. (2002). Nonstoichiometry and electrical transport in Sc-doped zirconia. Solid State Ionics.

[27] Holtan H., Mazur P., de Groot S.R. (1953). On the theory of thermocouples and thermocells. Physica.

[28] Ahlgren E.O., Poulsen F.W. (1995). Thermoelectric power of stabilized zirconia. Solid State Ionics.

[29] Fischer W. (1967). Die Thermokraft von kubisch stabilisiertem Zirkonoxid zwischen Sauerstoffelektroden. Z. Für Naturforschung A.

[30] Ruka R.J., Bauerle J.E., Dykstra L. (1968). Seebeck Coefficient of a (ZrO_2_)_0.85_ (CaO)_0.15_ Electrolyte Thermocell. J. Electrochem. Soc..

[31] Pizzini S., Riccardi C., Wagner V., Sinistri C. (1970). On the Thermoelectric Power of Stabilized Zirconia. Z. Für Naturforschung A.

[32] Yoo H.I., Hwang J.H. (1992). Thermoelectric behavior of single crystalline ZrO2(+8mo Y2O3). J. Phys. Chem. Solids.

[33] Ratkje S.K., Møller-Holst S. (1993). Exergy effeciency and local heat production in solid oxide fuel cells. Electrochim. Acta.

[34] Takehara Z.i., Kanamura K., Yoshioka S. (1989). Thermal Energy Generated by Entropy Change in Solid Oxide Fuel Cell. J. Electrochem. Soc..

[35] Ratkje S.K., Forland K.S. (1991). The Transported Entropy of Oxygen Ion in Yttria–Stabilized Zirconia. J. Electrochem. Soc..

[36] Kanamura K., Yoshioka S., Takehara Z.i. (1991). Dependence of Entropy Change of Single Electrodes on Partial Pressure in Solid Oxide Fuel Cells. J. Electrochem. Soc..

[37] Fischer K., Seume J.R. (2009). Location and Magnitude of Heat Sources in Solid Oxide Fuel Cells. J. Fuel Cell Sci. Technol..

[38] Nguyen Q.M., Takahashi T. (1995). Science and Technology of Ceramic Fuel Cells.

[39] Brodnikovskyi Y., McDonald N., Polishko I., Brodnikovskyi D., Brodnikovska I., Brychevskyi M., Kovalenko L., Vasylyev O., Belous A., Steinberger-Wilckens R. (2019). Properties of 10Sc1CeSZ-3.5YSZ(33-, 40-, 50-wt.%) Composite Ceramics for SOFC Application. Mater. Today Proc..

[40] Kjelstrup S., Bedeaux D. (2008). Non-Equilibrium Thermodynamics of Heterogeneous Systems.

[41] Forland K.S., Forland T., Ratkje S.K. (1988). Irreversible Thermodynamics: Theory and Applications.

[42] Kjelstrup S., Bedeaux D., Johannessen E., Gross J. (2010). Non-Equilibrium Thermodynamics for Engineers.

[43] Moore J.P., Graves R.S. (1973). Absolute Seebeck coefficient of platinum from 80 to 340 K and the thermal and electrical conductivities of lead from 80 to 400 K. J. Appl. Phys..

[44] Bedeaux D., Kjelstrup S. (2005). Heat Mass and Charge Transport and Chemical Reactions at Surfaces. Int. J. Thermodyn..

[45] Kabelac S., Siemer M., Ahrendts J. (2005). Thermodynamische Stoffdaten für Biogase. Forsch. Ingenieurwesen.

[46] International Organization for Standardization (2008). ISO/IEC Guide 98-3:2008: Uncertainty of Measurement—Part 3: Guide to the Expression of Uncertainty in Measurement (GUM).

[47] (2014). DIN EN 60584-1:2014-07. Thermoelemente_- Teil_1: Thermospannungen und Grenzabweichungen (IEC_60584-1:2013).

[48] Ahlgren E., Willy Poulsen F. (1994). Thermoelectric power of YSZ. Solid State Ionics.

